# Analysis of Patient-Dependent and Trauma-Dependent Risk Factors for Persistent Brachial Plexus Injury after Shoulder Dislocation

**DOI:** 10.1155/2018/4512137

**Published:** 2018-01-10

**Authors:** Olga Gutkowska, Jacek Martynkiewicz, Marek Stępniewski, Jerzy Gosk

**Affiliations:** Department of Traumatology, Clinical Department of Traumatology and Hand Surgery, Wroclaw Medical University, Wroclaw, Poland

## Abstract

Brachial plexus injuries (BPIs) caused by shoulder dislocation usually have a transient character and tend to resolve spontaneously. However, in some patients the symptoms can persist and require operative intervention. This work aims to determine the risk factors for persistent BPIs resulting from shoulder dislocation. The study comprised 73 patients (58 men, 15 women; mean age: 50 years) treated operatively between the years 2000 and 2016 for persistent BPIs resulting from shoulder dislocation. Patient age, gender, type of initial trauma, number of affected nerves, presence of accompanying injuries, and time interval from dislocation to its reduction were analysed. Elderly patients more often sustained multiple-nerve injuries, while single nerve injuries were more often observed in younger patients. Injury to a single nerve was diagnosed in 30% of the patients. Axillary nerve was most commonly affected. Fracture of the greater tuberosity of humerus coincided with total BPI in 50% of the cases. Longer unreduced period caused injury to multiple nerves. Analysis of our patient group against relevant literature revealed that persistent BPI after shoulder dislocation is more common in older patients. Injuries to ulnar and median nerves more often require operative intervention due to low potential for spontaneous recovery of these nerves.

## 1. Introduction

Injuries to the infraclavicular part of the brachial plexus constitute from one-fifth to one-third of all brachial plexus injuries (BPIs) [1–4]. They can be caused by penetrating injuries, blunt trauma, and fractures and dislocations around the shoulder joint, with the latter being responsible for about 7% of closed BPIs [5]. BPI caused by shoulder dislocation manifests usually as a transient sensory, motor, or mixed impairment affecting one or multiple nerves of the upper limb, which in the majority of the patients is resolved spontaneously or after a period of rehabilitation [3, 5–7]. However, in some patients, the symptoms tend to persist or the initial improvement in limb function ceases after a certain period of time. The severity of persistent BPIs resulting from shoulder dislocation varies from mild sensory impairment or local paraesthesia to complete paralysis of the upper extremity that requires timely operative intervention in order to obtain improvement in limb function. The aim of this work is to determine the risk factors for persistent and disabling BPI resulting from shoulder dislocation and to find out whether any certain group of dislocators requires enhanced surveillance for unrelenting neurologic deficit of the upper extremity.

## 2. Materials and Methods

The study comprised 73 patients (58 men, 15 women) treated operatively at our institution for BPI resulting from shoulder dislocation between the years 2000 and 2016. The patients were scheduled for operation on the basis of clinical examination and electromyography (EMG). Some patients had also magnetic resonance imaging of the brachial plexus performed (about 40% of the patients), although it was not considered indispensable in the presence of evident symptoms of brachial plexus injury and positive EMG examination results consistent with the clinical picture and positive history of shoulder dislocation. The following clinical scales were used to evaluate limb function: BMRC (British Medical Research Council) scale to assess motor function, BMRC scale modified by Omer and Dellon for the assessment of sensory function in the median and ulnar nerve distributions, and Highet's classification to assess sensory function in the areas innervated by the axillary, radial, and musculocutaneous nerves. Tinel-Hoffman sign was examined in all of the patients. Limb function was evaluated at least twice before operation in all of the patients: for the first time at the initial visit and again on admission to the hospital, after a mean period of 3 months. All patients were examined by the senior author (J. G.), who also performed all of the operative procedures, at their initial visit to our clinic and again before the operation, which reduces the risk of bias resulting from different examination techniques. In several patients, in whom spontaneous, satisfactory improvement of nerve function was observed at their second visit, conservative management was continued by neurologists on the outpatient basis close to their places of residence. The other patients underwent operative treatment after a mean period of 9.2 months after dislocation (range, 1–84 months). Such factors as patient age, gender, type of initial injury causing shoulder dislocation, the number of affected nerves (injuries to a single nerve or to multiple nerves), the presence and type of accompanying injuries, and time interval from dislocation to its reduction were analysed.

Statistical analysis of the results was conducted with the use of STATISTICA software version 13 (TIBCO Software Inc.). Categorical data was analysed using the chi-square test with Yate's correction. The distribution of two and more than two independent samples was compared through Mann–Whitney *U* test and Kruskal-Wallis test, respectively. The statistical significance was defined as *p* value ≤ 0.05.

## 3. Results

Seventy-three patients were treated operatively for BPI resulting from shoulder dislocation. Males predominated in the group (79,5%) and were affected almost 4 times more often than females (F : M = 1 : 3.87). Mean patient age was 50 years and 1 month (range: 19 years and 3 months to 75 years and 6 months); it was slightly higher for females, 52 years and 1 month, than for males, 49 years and 7 months. The right side was affected in 40 patients (55%) and the left side in 33 patients (45%). Injury to a single nerve was diagnosed in 30% of the patients (axillary nerve: 17 cases, ulnar nerve: 4 cases, and median nerve: 1 case) and to multiple nerves in 70% of the patients (51 persons). The mean age of the patients who manifested symptoms of injury to a single nerve was 43 years and 7 months, while the patients diagnosed with multiple-nerve injury were older, with a mean age of 52 years and 11 months. Shoulder dislocation was a result of a simple fall considered low-energy trauma in half of the patients (37 patients) and high-energy trauma in 28 patients (38%) (fall from a height: 21 patients and motor vehicle accident: 7 patients). In eight patients who suffered industrial or occupational accidents, it was impossible to classify the type of initial trauma in either of the two categories and these patients were excluded from the analysis in relation to the type of initial trauma. High-energy mechanism caused single nerve injury in 14 patients and multiple-nerve injuries in an equal number of patients. In the group of nerve injuries resulting from low-energy trauma (simple fall) injuries to multiple nerves predominated and were observed in 86.5% of the patients. Out of 7 patients who sustained shoulder dislocation as a result of motor vehicle accident, 6 had single nerve injuries, including 4 cases of axillary nerve palsy. In total, 219 nerves were affected in 73 patients (the mean number of nerves involved was 3). Injuries to a single nerve made up 10% of the total number of injured nerves. The summary of demographic data of patients included in this study is presented in Table 1. Detailed demographic data of patient subgroups is presented in Table 2.

Axillary nerve was most commonly affected, both when the total number of nerves is considered and as a single nerve. Axillary nerve injuries constituted almost one-fourth of all observed nerve injuries (24.7%) and more than 77% of injuries to single nerves. Only in 18 patients (24.7%) there were no symptoms of axillary nerve injury. The second most commonly affected nerve was the ulnar nerve, both as a single nerve (18%) and when the total number of nerves is considered (23%). Injuries to the musculocutaneous nerve were the rarest and were found in less than 10% of the cases. The radial and musculocutaneous nerves were injured only as a part of multiple-nerve injury. No single nerve injuries to these nerves were observed. The numbers of injuries to particular nerves are presented in Table 3 in descending order of frequency.

In 41 patients (56%), it was possible to determine the time interval from dislocation to its reduction. In all seven patients, in whom dislocated shoulder remained unreduced for more than 6 hours (range: 7–48 hours), injuries to multiple nerves were found. In the remaining cases, injuries to many nerves were found in 62% of the patients and to a single nerve in 38% of the cases. Injuries to multiple nerves were encountered more often in patients older than 60 years of age (68% of all multiple-nerve injuries), while 72% of all single nerve injuries were found in patients younger than 60 years. The correlation between the number of injured nerves and patient age was found to be statistically significant (*p* = 0,004).

In 33 patients (45%), accompanying injuries were found, except for neurologic deficit (Table 4). The most common accompanying injury was fracture of the greater tuberosity of humerus (GTF), found in 22 patients. Rotator cuff tear (RCT) was observed rarer (in 7 cases) and the mean age of these patients was higher than the mean patient age for the whole patient group (54 years and 8 months versus 50 years and 1 month), with all RCTs occurring in patients older than 48 years. RCT coincided with isolated injury to axillary nerve in more than half of the cases (57%), creating the so-called unhappy or terrible triad of the shoulder. Moreover, 4 proximal humeral fractures were diagnosed, which were associated with injury to the axillary nerve in 3 cases and to the radial nerve in 2 cases. One-third of the patients with accompanying injuries at the time of the initial trauma were found to have injuries to a single nerve. Injuries were caused by high-energy trauma in half of the patients with accompanying injuries and by low-energy trauma in the other half. No accompanying vascular injuries were observed in our patient group.

Total brachial plexus palsy defined as affection of all 5 major nerves of the upper extremity (axillary, radial, ulnar, median, and musculocutaneous nerves) was found in 20 patients (27%). The mean age of these patients was comparable with the mean age for the whole patient group (50 years and 3 months). Men were found to have total brachial plexus palsy 3 times more often than women. Total palsy resulted from high-energy injuries as often as from low-energy injuries (9 versus 8 patients), with 3 exclusions. Affection of all 5 major nerves of the upper extremity was associated with the presence of greater tuberosity fracture in 55% of the patients. At the same time, 50% of GTFs coexisted with the total palsy.

The results of statistical analysis revealed that injury to multiple nerves was observed in the group of patients older than 60 years of age statistically significantly more often than in other age groups (*p* = 0.004). The analysis confirmed also the relationship between the energy of the initial trauma and the number of affected nerves. High-energy trauma caused single nerve injury more often, with statistical significance (*p* = 0.006, with the exclusion of eight cases with uncertain energy of the initial trauma). The analysis did not confirm the relationship between the number of injured nerves and the presence/absence of accompanying injuries, patient gender, or body side.

In all of the operated-on patients, injury to the infraclavicular part of the brachial plexus at the level of cords and nerves was observed. No signs of trauma to the supraclavicular brachial plexus, including nerve root avulsion, were found. Fibrous scar tissue around neural elements causing their compression was a constant finding observed intraoperatively in all of the patients. In 8.2% of the patients, intraneural fibrosis was diagnosed with the use of loupe magnification or operating microscope. Complete nerve disruption requiring repair with sural nerve grafting was seen in only two patients (2.7%) and in both cases it was a rupture of the axillary nerve. It was determined that shoulder dislocation caused mostly I–III degree injury according to Sunderland (neurapraxia or axonotmesis according to Seddon).

Operative treatment allowed for improvement of patients' upper limb function in the majority of the cases. No serious complications occurred intra- and postoperatively. The best results were observed for injuries to the musculocutaneous and radial nerves (satisfactory recovery of function in 93%–100% of the cases), while the worst results were found in patients who suffered injury to the ulnar nerve (good recovery of function in only half of the cases) and the axillary nerve (no recovery in 1/5 of the cases). Injury to a single nerve carried worse prognosis for recovery after operative treatment than injury to multiple nerves. The above-cited data refers to a subgroup of 33 patients who completed a 2-year follow-up. Detailed information regarding the results of operative treatment obtained in this patient group was presented in another publication [8].

## 4. Discussion

Shoulder dislocation is the most common dislocation of a major joint in humans with the annual incidence of 26.9 per 100,000 per year [9]. Dislocating humeral head can cause damage to bony, fibrous, ligamentous, vascular, and neural structures. Injuries to nerves are among the rarest, with the reported incidence ranging from 3% up to 55% in the EMG study by Toolanen et al. on patients older than 40 years of age [9–24]. However, at the same time, nerve injuries are associated with the risk of persistent compromise of limb function. Neurologic injuries resulting from shoulder dislocation involve mainly infraclavicular part of the brachial plexus and are usually characterised by neurapraxia and rarer axonotmesis [12, 24–27]. According to Adams and Duchen, axonotmesis is observed when the strength of stretch injury exceeds 10–20% of the tissue elastic limit [28].

BPIs result from three major mechanisms: (1) traction of neural structures by humeral head when it leaves the glenoid, (2) mechanical compression exerted by humeral head on elements of the brachial plexus, and (3) compression on neural elements by haematoma and resulting fibrous tissue [29]. The majority of neurologic injuries have transient character and can be treated conservatively. The reported rates of spontaneous recovery are high: 75% immediately after reduction in the study by Perron et al., 89% of patients who had nerve injury diagnosed electromyographically in the study by Visser et al., and 100% in the studies by Gumina and Postacchini and Saragaglia et al. [13, 19, 20, 30]. However, the rate of spontaneous recovery of neurologic injuries decreases with increasing patient age, especially above 40–50 years of age [6, 18]. In such cases no recovery is observed or recovery ceases after initial improvement. Pasila et al. reported lack of complete recovery in 15 out of 44 patients (34%) with neurologic deficit after one year of conservative treatment, while Visser et al. found no improvement of function in 12.5% of axillary nerve injuries, in both EMG and clinical examination [18, 30]. In the study by Johnson and Bayley, the reported incidence of residual reduction of limb mobility was as high as 60% [31]. In such cases, the symptoms of BPI tend to persist causing compromise of the upper limb function. These patients should be considered candidates for operative intervention after a mean observation period of 6 months [2, 6, 32–37]. In clinical practice, the mean time to operation in cases with no recovery is usually longer due to organizational reasons and patient-dependent factors. In our patients, the mean time from dislocation to operation was 9.2 months, which is comparable to the study by Wehbe et al., in which 33 patients with axillary nerve injury after shoulder dislocation were subjected to operative treatment after a mean period of 10 months [29]. This time period was long enough to determine indications for surgical treatment and at the same time, for most patients, it was in line with the guidelines for operative intervention on nerves.

It is worth noticing that the mean age of patients with neurologic deficit resulting from shoulder dislocation, including our patient group, is significantly higher than that of patients sustaining isolated shoulder dislocation in the majority of the studied groups (e.g., mean age in patients with isolated dislocation: Perron et al.: 34.3 years, Atef et al.: 35.2 years, te Slaa et al.: 39 years, and Lill et al.: 41 years; mean age in patients with BPI after shoulder dislocation: Hems and Mahmood: 52 years, Robinson et al.: 51.5 years, and our study: 50 years) [9, 10, 14, 19, 21, 38]. High patient age is considered one of the major risk factors for neurologic complications of shoulder dislocation, especially above 50 years of age [9, 11, 18, 22, 24, 34]. According to Visser et al., the probability of neurologic injury increases with a factor of 1.3 per ten-year period and older age is also associated with slower and incomplete recovery [30].

The role of energy of the initial trauma causing shoulder dislocation has also been raised. According to Robinson et al., the risk of neurologic deficit resulting from shoulder dislocation is higher in patients who suffered low-energy injury [9]. Conversely, Yeap et al. and Pasila et al. found higher energy of the initial trauma to be connected with elevated risk of neural complications [17, 18, 24]. The structure of initial injuries causing shoulder dislocation was comparable in patients treated conservatively for neurologic deficit after shoulder dislocation and in our patient group requiring operative intervention. According to different authors, the initial injury was the result of a simple fall in 43–67%, a fall from a height in 11–23%, and a motor vehicle accident (MVA) in 10–12.5% of the cases [9, 10, 38]. Among our patients these numbers were 50%, 28.8%, and 17.1%, respectively. Considering similar distribution of initial injury mechanisms among conservatively treated patients, studied by other authors and in our patient group requiring operative intervention, it can be concluded that no particular mechanism of initial trauma predisposes patients to nerve injury resistant to conservative treatment. High-energy trauma may cause more severe damage to neural elements but it is encountered mainly in younger patients with a higher regenerative capacity. On the other hand, a simple fall causes less severe injuries but patients' older age limits their potential for spontaneous regeneration.

Long period of time from dislocation to its reduction was found to increase the risk of neural complications in the studies by Pasila et al. and Yeap et al. [18, 24]. Duration of dislocation was possible to determine in more than half of our patients and it was longer than 6 hours in 17% of them. All of these patients suffered injury to multiple nerves.

As far as gender distribution is concerned, in almost all studied groups, comprising patients with isolated shoulder dislocation, neurologic deficit treated successfully with conservative method, or persistent neurologic injury requiring operative intervention, pronounced predomination of men was observed [24, 29, 37, 39, 40].

The distribution of nerve injuries in our patient group as presented in Table 3 is similar to the results obtained by Hems and Mahmood, with the most frequent involvement of axillary and ulnar nerves, and with median and radial nerves occupying the 3rd and 4th position [38]. Particular nerves have different potential for spontaneous recovery depending on the distance to the effector, with the highest recovery rates observed in the study by Hems and Mahmood for the axillary, musculocutaneous, and radial nerves and inferior results for the median and ulnar nerves (incomplete or no recovery), especially regarding intrinsic muscles of the hand [38]. The time periods required for obtaining recovery after operative intervention in the study by Lam et al. also varied from 14 months for elbow flexion to 25 months for wrist flexion [34]. Functional improvement in the interossei can be expected, if at all present, after a substantially longer period of time (18–36 months, according to Leffert and Seddon) [5]. This may explain why ulnar and median nerve injuries were the 2nd and 3rd most common nerve injuries in our operatively treated patient group, while they occupied the last two places in conservatively managed groups studied by de Laat et al. and Visser et al. [12, 30]. Although injuries to these nerves are relatively rare, their low potential for spontaneous recovery makes them more likely to require operative intervention. Similarly, musculocutaneous nerve injuries reported to be the 2nd most common nerve injury in the two aforementioned patient groups occupied the last place among our patients thanks to relatively short course of this nerve to the effector muscle and its high potential for recovery [12, 30].

In the study by Robinson et al. on 3633 patients with shoulder dislocation, the incidence of neurologic deficit was 13.5%, out of which 5.8% was isolated, 5.7% combined with GTF, and 2.1% combined with RCT. Similar numbers were reported for the patient group studied by Atef et al., in which the incidence of neurologic deficit was 15.8% of the patients, out of which only 3.3% was isolated (about 1/5 of all neurologic complications) and 6.25% was associated with either RCT or GTF [10]. In the study by Hems and Mahmood, GTF/RCT accompanied BPI in 31% of the cases [38]. According to Robinsons et al., the presence of RCT/GTF is associated with increased risk of neurologic deficit (RR, 1.9) [9]. Coexistence of neurologic deficit and other accompanying injuries was found to be connected with higher patient age, over 45 years according to Johnson and Bayley and over 60 years according to Robinson et al. [9, 31]. Both in our study and in the study by Atef et al., the mean age of the patients suffering from GTF and nerve injury was lower than the mean age of the whole patient group, while the mean age of patients suffering from RCT and nerve injury was higher than the mean age of the whole group (mean patient age, RCT versus GTF: 53.9 years versus 32.8 years in the study by Atef et al. and 54 y and 8 m versus 48 y and 8 m in our study). In the aforementioned studies on conservatively treated patients, neurologic deficit coexisting with other injuries was more common than isolated deficit, contrary to our patients requiring operative intervention. A suggestion can be made that isolated neurologic injury more often tends to be persistent.

Fracture of the greater tuberosity of the humerus was the most common accompanying injury in our patient group. Combination of GTF and neurologic deficit was observed in 30% of our patients and in almost 42% of patients studied by Robinson et al. but only in 6.25% of the cases in the study by Atef et al. [9, 10]. In our study the presence of GTF was associated with injury to all long nerves of the brachial plexus in more than half of the cases. Similar observation was made by Robinson et al., who found that in their patient group “patients with multiple-nerve lesions were more likely to have a residually displaced greater tuberosity fracture” [9]. In some case reports GTF also coincided with total brachial plexus palsy [41, 42]. According to Visser et al., the presence of GTF doubled the incidence of nerve injury, adversely affecting its severity and the number of injured nerves [30]. Hems and Mahmood advocate early repair of GTF associated with BPI in order to optimize the overall result [38].

RCT accompanying nerve injury was observed in 6.25–15% of all patients with neurologic deficit in different studies [9, 10, 30, 43]. In our patients, the incidence of the unhappy triad was equal to that reported by Visser et al. (9%) [30]. In the abovementioned studies, the mean age of the patients with coexisting RCT was higher than those with neurologic deficit alone, which was also confirmed in our study. The mean age of six patients treated for unhappy triad of the shoulder by Simonich and Wright was 57 years [27]. All cases with RCT in the study by Atef et al. were observed in patients older than 45 years and the severity of RCT increased with patient age [10]. None of our patients suffering from neurologic deficit and RCT was younger than 48 years. In our study, axillary nerve injury accompanied RCT in 6 out of 7 cases and in 4 cases it was a single nerve injury. Similarly, in the EMG/USG study by Toolanen et al., half of the patients diagnosed with RCT after shoulder dislocation had accompanying injury to axillary nerve alone [22].

Injuries to a single nerve were found more often in younger patients and resulted from higher energy trauma. These findings are fully consistent with the observations made by Robinson et al. [9]. In our patient group, injuries to a single nerve were observed in 30% of the patients, rarer than the numbers reported by most authors (Yeap et al.: 51%, Visser et al. [30]: 51%, Robinson et al.: 90,5%, and Atef et al.: 100%) but similar to the study by de Laat in which solitary axillary nerve palsy accounted for 32% of the cases and by Visser et al. [44], in which single nerve injuries were encountered in 30% of the patients [9, 10, 12, 24, 30, 44]. Lower percentage of single nerve injuries in patients requiring operative treatment in comparison to conservatively treated patients may result from high potential for spontaneous recovery of the axillary nerve, which is the most commonly injured single nerve [9, 11, 12, 24, 38, 39, 44]. The incidence of total BPI was similar among our patients and the study group described by Hems and Mahmood (27% and 25%, resp.) [38].

The well-established fact that the axillary nerve is the most commonly injured nerve as a result of shoulder dislocation has also been confirmed in our study, when both the total number of nerve injuries and the number of injuries to a single nerve are considered [9, 11, 12, 24, 38, 39, 44]. Literature data confirms that elevated risk of axillary nerve injury is associated with increasing patient age, longer duration of dislocation, and higher energy of the initial trauma [25]. Robinson et al. found the demographic features of patients with axillary nerve injury to be comparable with those who had isolated dislocation [9]. Other nerves were rarely affected as a solitary nerve injury in our patient group, similarly to the literature data [9].

Only a fraction of patients with BPI after shoulder dislocation (up to 15% in the study by Hems and Mahmood) require operative intervention [38]. Depending on the severity of neural lesions, BPI may require neurolysis, which is sufficient in the majority of the cases or nerve grafting in case of a complete nerve rupture, which is rare in BPI caused by shoulder dislocation (axillary nerve disruption was observed in 2.7% of our patients and in 2.4% of the patients treated by Hems and Mahmood and always resulted from high-energy injury) [38].

Timely operative intervention turned out to be capable of improving limb function after brachial plexus injury in the majority of the patients, with external neurolysis of the infraclavicular part of the brachial plexus being the procedure of choice. Worse postoperative recovery observed in single nerve injuries in comparison to multiple-nerve injuries may be explained by higher occurrence of the former as a result of high-energy trauma. Similarly to observations made by other authors, in our patient group the highest rates of spontaneous return of function were observed for the musculocutaneous and radial nerves [38, 39]. Due to long distance to the effector muscles, ulnar nerve injuries demonstrated delayed or incomplete recovery, which confirms previously published literature data [5, 34, 38]. Despite generally high potential for recovery of the axillary nerve and its short course, 20% of our patients with an injury to this nerve had poor results after operative intervention. Similar percentages for axillary nerve injuries were reported in the studies by Wehbe et al. and Travlos et al., the latter of whom found isolated axillary nerve injuries to have the worst prognosis for recovery [29, 37]. In neither of the two cases of the axillary nerve rupture in our patients, return of its function was obtained. In patients who are seen a long time after the initial injury or in whom neurolysis turned out unsuccessful, neurotisation or tendon and muscle transfers should be considered.

The major limitation of this study is the absence of a control group consisting of conservatively treated patients, in whom BPI resulting from shoulder dislocation recovered spontaneously or after a period of rehabilitation. This limitation is a consequence of our institution being a tertiary referral centre specializing in brachial plexus surgery, to which patients are referred only after failed conservative treatment at other institutions. Out of the presented patient group, only two patients were initially seen at our department just after dislocation, and all of the other patients were referred to our institution from other primary and secondary medical care centres from all over the country.

## 5. Conclusions

Comparing the data of our patient group with the literature dedicated to BPI resulting from shoulder dislocation, it can be stated that such characteristics as mean patient age, gender, frequency of particular patterns of initial trauma causing shoulder dislocation, or presence of accompanying lesions do not differ between patients requiring operative intervention for brachial plexus injury and those with spontaneous recovery of nerve function.

Vigilance and systematic control are, therefore, recommended in all patients manifesting symptoms of neurologic deficit after shoulder dislocation. While no typical characteristics of a “patient at risk” can be listed, there are several points that can be helpful in evaluation:

- Older patients are more likely to suffer neurologic complications after shoulder dislocation and have lower chances for spontaneous recovery.
- The ulnar and median nerve injuries have the lowest potential for spontaneous recovery and tend to be persistent.
- Multiple-nerve injury should be suspected in patients, in whom shoulder dislocation remained unreduced for more than 6 hours.
- Fracture of the greater tuberosity of humerus often coincides with injury to all long nerves of the brachial plexus.
- Multiple-nerve rather than single nerve injury should be sought for in an elderly patient who sustained shoulder dislocation as a result of a simple fall.
- Younger patients after high-energy trauma are more likely to suffer single nerve injury; in the majority of the cases the axillary nerve is affected.

Nonoperative treatment is a commonly recommended approach in infraclavicular BPIs resulting from shoulder dislocation [5, 12, 26, 37, 38, 45, 46]. However, some patients will not benefit from conservative management. These patients need to be identified and referred to specialist centres for timely operative intervention. Prolonging the observation period by more than 6 months in the absence of symptoms of recovery in a patient with disabling nerve injury cannot be justified and reduces the patient's chances for regaining useful limb function, even if operative treatment is finally performed.

## Figures and Tables

**Table 1:** The summary of demographic data of patients with persistent brachial plexus injury after shoulder dislocation requiring operative intervention.

Mean patient age (range)	50 yr and 1 mo (19 yr and 3 mo–73 yr and 6 mo)

Mean time from dislocation to operation (range)	9.2 mo (1–84 mo)

Gender	
Male	58
Female	15

Side	
Right	40
Left	33

Mechanism of injury	
Low energy	37
High energy	28
Not specified	8

Type of brachial plexus injury	
Single nerve	22
Partial	31
Complete	20

Accompanying injuries	
Absent	40
Present	33
GTF	22
RCT	7
HF	4

Time from dislocation to reduction	
Not known	32
Known	41
≤6 hours	34
>7 hours	7

yr: year; mo: month; GTF: fracture of the greater tuberosity of humerus, RCT: rotator cuff tear, and HF: humeral fracture.

**Table 2:** Detailed demographic data of patients with persistent brachial plexus injury after shoulder dislocation requiring operative intervention.

Injury category	No. of pts (%)	Mean age	M : F	Simple fall	Fall from a height	MVA	Other	R : L
Isolated dislocation	40 (55%)	50 y and 5 m	4 : 1	23	10	4	3	20 : 20
Dislocation + accomp. injury	33 (45%)	49 y and 3 m	3.7 : 1	14	11	3	5	20 : 13
Dislocation + GTF	22 (30%)	48 y and 8 m	5.5 : 1	10	8	2	2	14 : 8
Dislocation + RCT	7 (9.6%)	54 y and 8 m	2.5 : 1	4	3	0	0	4 : 3
Dislocation + HF	4 (5.4%)	47 y and 7 m	3 : 1	0	0	1	3	2 : 2
Single nerve injury	22 (30%)	43 y and 7 m	5.5 : 1	5	8	6	3	11 : 11
Complete BPI	20 (27%)	53 y and 3 m	3 : 1	8	9	0	3	12 : 8

Total	73	50 y and 1 m	3.9 : 1	37 (51%)	21 (29%)	7 (9%)	8 (11%)	40 : 33

No.: number; pts: patients; M: males; F: females; MVA: motor vehicle accident; R: right; L: left; y: years; m: months; accomp.: accompanying; GTF: fracture of greater tuberosity of humerus; RCT: rotator cuff tear; HF: humeral fracture; BPI: brachial plexus injury.

**Table 3:** Injury to particular nerves in the studied patient group.

Nerve	No. of injured nerves (%)	No. of SNI^*∗*^ (%)	GHD + GTF (as SNI^*∗*^)	GHD + RCT (as SNI^*∗*^)	GHD + HF (as SNI^*∗*^)	GHD + accomp. inj/isolated GHD (as SNI^*∗*^)	Mean age^*∗∗*^ in SNI^*∗*^
Axillary	54 (24,7%)	17 (7,7%)	19 (4)	6 (4)	3 (1)	28/26 (9/8)	44 y and 11 m
Ulnar	51 (23,3%)	4 (1,8%)	17 (1)	3 (0)	0 (0)	20/31 (1/3)	37 y and 11 m
Median	48 (21,9%)	1 (0,5%)	18 (0)	3 (0)	1 (1)	22/26 (1/0)	43 y and 6 m
Radial	45 (20,6%)	0	18 (0)	3 (0)	2 (0)	23/22 (0/0)	-
Musculocutaneous	21 (9,5%)	0	11 (0)	1 (0)	1 (0)	13/8 (0/0)	-

Total	219 (100%)	22 (10%)	83 (5)	16 (4)	7 (2)	106/113 (11/11)	

^*∗*^SNI: single nerve injury. ^*∗∗*^Mean age for all patients with single nerve injuries: 43 y and 7 m; all patients with multiple nerve injuries: 52 y and 11 m; and all patients: 50 y and 1 m; No.: number; GHD: glenohumeral dislocation; GTF: fracture of the greater tuberosity of humerus; RCT: rotator cuff tear; HF: humeral fracture; accomp.: accompanying; inj: injury; y: years; m: months.

**Table 4:** Frequency and combination of nerve lesions in isolated dislocations and in combination with GTF, RCT, and HF (no. of cases).

Initial trauma	Single nerve injury	Multiple-nerve injury (2–4 nerves)	Complete brachial plexus palsy (5 nerves)
Isolated dislocation	11 (A-8, U-3)	21	8
Dislocation + accomp. injury	11 (A-9, M-1, U-1)	10	12
Dislocation + GTF	5 (A-4, U-1)	6	11
Dislocation + RCT	4 (A-4)	2	1
Dislocation + HF	2 (A-1, M-1)	2	0

GTF: fracture of the greater tuberosity of humerus; RCT: rotator cuff tear; HF: humeral fracture; no.: number; accomp.: accompanying; A: axillary nerve; U: ulnar nerve; M: median nerve.
